# Public health round-up

**DOI:** 10.2471/BLT.17.010417

**Published:** 2017-04-01

**World Hearing Day**

A child undergoing an ear examination in Madagascar. The theme of this year’s World Hearing Day on 3 March: “Action for hearing loss: make a sound investment” draws attention to the economic impact of hearing loss.

**Figure:**
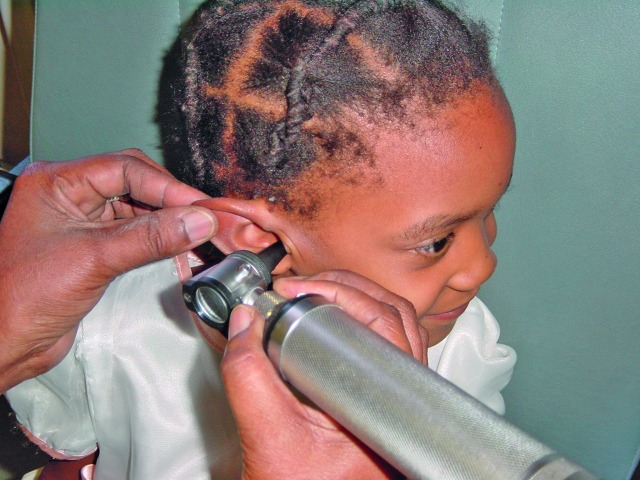
CBM/P van Hasselt

## She Decides conference

More than 400 participants from 50 countries, including 20 government ministers, gathered in Brussels on 2 March to pledge their continued support for the sexual and reproductive health and rights of women worldwide. 

The event was co-organised by the governments of Belgium, Denmark, the Netherlands and Sweden following the launch of the She Decides initiative by the Minister for Foreign Trade and Development Cooperation of the Netherlands, Lilianne Ploumen, on 24 January.

She Decides is a new global initiative that supports women’s rights to decide freely whether, when and with whom they have children, and how many children to have. 

In February, a group of experts called on the international community to protect women’s rights to the highest attainable standards of sexual and reproductive health, to safeguard progress made to date, and to sustain and expand national women’s health programmes.

In their statement, members of the Scientific and Technical Advisory Group and the Gender and Rights Advisory Panel of the UNDP/UNFPA/UNICEF/WHO/World Bank Special Programme of Research, Development and Research Training in Human Reproduction, said: 

“We … are deeply concerned that current global trends will restrict access to life-saving sexual and reproductive health services and information for women and girls, especially those most in need, and [we] fear that the significant gains made over the past three decades will be compromised.

“It is critical to ensure access to comprehensive sexual and reproductive health services … so that no one is left behind,” the group said.

http://www.who.int/life-course/news/events/she-decides-conference

## Depression: new estimates

Globally the number of people with depression was estimated to exceed 300 million in 2015, according to a new World Health Organization (WHO) report, *Depression and other common mental disorders: global health estimates*, released on 23 February.

In addition, over 250 million people suffer from a range of anxiety disorders, according to the report.

Rates of depression and anxiety disorders vary across countries and regions, as well as by age and sex, as shown by disaggregated findings presented in the report.

The consequences of these disorders in terms of lost health are huge. Depression is ranked by WHO as the single largest contributor to global disability, accounting for 7.5% of all years lived with disability in 2015. Anxiety disorders are ranked as the sixth largest contributor to global disability, accounting for 3.4% of all years lived with disability in 2015.

Depression is a major contributor to suicide deaths, which number close to 800 000 per year globally.

The report was released ahead of World Health Day on 7 April, the theme of which is “Depression: let’s talk”.

http://www.who.int/mental_health/management/depression/prevalence_global_health_estimates

## Chemical weapons response 

WHO, its partners and local health authorities activated an emergency response plan last month to treat people who have been exposed to highly toxic chemicals, following reports that chemical weapons had been used in East Mosul in Iraq.

Between 1 and 3 March, 12 patients received treatment at a referral hospital in the Iraqi city of Erbil, which is about 80 km away from Mosul, for respiratory symptoms and blistering associated with exposure to a blister agent, according to local health authorities. Four of the patients showed severe signs of these symptoms.

A blister agent is a chemical compound that causes severe skin, eye and mucosal pain and irritation.

WHO and its partners were working with the health authorities in Erbil to provide support in managing these patients.

As part of a chemical weapons contingency plan, WHO experts have trained more than 120 clinicians and provided them with equipment to safely decontaminate and stabilize patients before they are referred to hospitals for further care.

WHO is extremely alarmed by the use of chemical weapons in Mosul, where civilians are already suffering as a result of the ongoing conflict.

The use of chemical weapons is a war crime and is prohibited in a series of international treaties.

http://www.emro.who.int/media/news/who-responds-to-reported-use-of-chemical-weapons-agents-in-east-mosul-iraq.html

## Child health risks 

More than one in four deaths of children aged under five years are attributable to unhealthy environments.

Every year, environmental risks – such as indoor and outdoor air pollution, second-hand smoke, unsafe water, lack of sanitation, and inadequate hygiene – take the lives of 1.7 million children aged under five years, according to two WHO reports released last month.

The first report, *Inheriting a sustainable world? Atlas on children’s health and the environment* reveals that a large percentage of the most common causes of death among children aged one month to five years – diarrhoea, malaria and pneumonia – are preventable by interventions known to reduce environmental risks, such as access to safe water and clean cooking fuels.

"A polluted environment is a deadly one – particularly for young children," said Dr Margaret Chan, WHO Director-General. "Their developing organs and immune systems, and smaller bodies and airways, make them especially vulnerable to dirty air and water."

Harmful exposures can start in the mother’s womb and increase the risk of premature birth.

Additionally, when infants and pre-schoolers are exposed to indoor and outdoor air pollution and second-hand smoke they have an increased risk of pneumonia in childhood, and a lifelong increased risk of chronic respiratory diseases, such as asthma. Exposure to air pollution may also increase their lifelong risk of heart disease, stroke and cancer.

A companion report, *Don't pollute my future! The impact of the environment on children's health*, provides a comprehensive overview of the environment’s impact on children’s health, illustrating the scale of the challenge.

http://www.who.int/ceh/publications/don-t-pollute-my-future

http://www.who.int/ceh/publications/inheriting-a-sustainable-world

**Cover photo**

This three-year-old boy and his family fled from gang violence in Honduras and found safety over the border in Mexico, living in a shelter that is funded by the United Nations High Commissioner for Refugees (UNHCR) in the south-eastern town of Tenosique.

**Figure:**
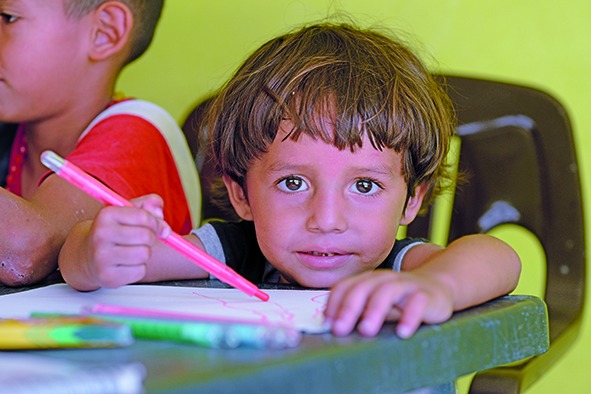
© UNHCR/Sebastian Rich

## Resistant pathogens 

WHO published a list of antibiotic-resistant “priority pathogens” – a catalogue of 12 bacteria – to guide and promote research and development of new antibiotics to address growing global resistance to antimicrobial medicines.

Mycobacterium *tuberculosis* was not included in the list because – although it has become increasingly resistant to first-line treatments in recent years – it is already accepted broadly as a global priority that is targeted by dedicated tuberculosis programmes.

Other bacteria that were not included, such as streptococcus A and B and chlamydia, have low levels of resistance to existing treatments and do not currently pose a significant public health threat.

The WHO list, released on 27 February, is divided into three categories according to the urgency of need for new antibiotics: critical, high and medium priority.

The most critical group of all includes multidrug-resistant bacteria that pose a particular threat in hospitals, nursing homes and among patients whose care requires devices, such as ventilators and intravenous catheters.

The second and third tiers in the list – the high and medium priority categories – contain other increasingly drug-resistant bacteria that cause more common diseases, such as gonorrhoea (caused by Neisseria *gonorrhoea *resistant to fluoroquinolones and cephalosporins) and food poisoning (Salmonella* spp *resistant to fluoroquinolones).

http://www.who.int/medicines/publications/global-priority-list-antibiotic-resistant-bacteria

## R&D for tuberculosis

WHO reaffirmed the critical need for research and development of new antibiotics to tackle the threat of drug-resistant tuberculosis on 28 February.

"Addressing drug-resistant tuberculosis research is a top priority for WHO and for the world," said Dr Margaret Chan, WHO Director-General. "More than US$ 800 million per year is currently necessary to fund badly needed research into new antibiotics to treat tuberculosis."

The multidrug-resistant tuberculosis (MDR–TB) public health crisis continues: there were an estimated 580 000 cases and 250 000 related deaths in 2015. Only 125 000 were started on treatment, and just half of those people were cured.

Only two new antibiotics to address MDR–TB have completed Phase IIB trials in the past 50 years. Both are still in Phase III trials, and more funding will be required to complete the process and to develop other effective treatment regimens.

Drug-resistant tuberculosis and research will be major themes at the WHO Ministerial Conference on tuberculosis planned in Moscow in November 2017. These issues will also be on the agenda at the United Nations General Assembly high-level meeting on tuberculosis in 2018. 

http://www.who.int/mediacentre/news/releases/2017/drug-resistant-tb

**Looking ahead**

25–26 April – United Nations International Conference on Sustainable Development, London, United Kingdom of Great Britain and Northern Ireland

10–12 May – High-Level eHealth Conference, Malta. This year’s theme is “data for health: the key to personalized sustainable care”

22–31 May – 70th World Health Assembly, Geneva, Switzerland

31 May – World No Tobacco Day. This year’s theme is “tobacco: a threat to development”

